# Effects of *TM6SF2* E167K on hepatic lipid and very low-density lipoprotein metabolism in humans

**DOI:** 10.1172/jci.insight.144079

**Published:** 2020-12-17

**Authors:** Jan Borén, Martin Adiels, Elias Björnson, Niina Matikainen, Sanni Söderlund, Joel Rämö, Marcus Ståhlman, Pietari Ripatti, Samuli Ripatti, Aarno Palotie, Rosellina M. Mancina, Antti Hakkarainen, Stefano Romeo, Chris J. Packard, Marja-Riitta Taskinen

**Affiliations:** 1 Department of Molecular and Clinical Medicine, Institute of Medicine, University of Gothenburg, Gothenburg, Sweden.; 2 Wallenberg Laboratory for Cardiovascular and Metabolic Research, Department of Cardiology, Sahlgrenska University Hospital, Gothenburg, Sweden.; 3 Research Program for Clinical and Molecular Metabolism, Faculty of Medicine, University of Helsinki, Helsinki, Finland.; 4 Endocrinology, Abdominal Center, Helsinki University Hospital, Helsinki, Finland.; 5 Institute for Molecular Medicine Finland, Helsinki Institute of Life Science (HiLIFE), University of Helsinki, Helsinki, Finland.; 6 Broad Institute of the Massachusetts Institute of Technology and Harvard University, Cambridge, Massachusetts, USA.; 7 Department of Public Health, Clinicum, Faculty of Medicine, University of Helsinki, Helsinki, Finland.; 8 Helsinki and Uusimaa Hospital District Medical Imaging Center, Radiology, Helsinki University Hospital, University of Helsinki, Finland.; 9 Institute of Cardiovascular & Medical Sciences, University of Glasgow, Glasgow, United Kingdom.

**Keywords:** Hepatology, Metabolism, Lipoproteins

## Abstract

Nonalcoholic fatty liver disease (NAFLD) is characterized by hepatic lipid accumulation. The transmembrane 6 superfamily member 2 (*TM6SF2*) E167K genetic variant associates with NAFLD and with reduced plasma triglyceride levels in humans. However, the molecular mechanisms underlying these associations remain unclear. We hypothesized that *TM6SF2* E167K affects hepatic very low-density lipoprotein (VLDL) secretion and studied the kinetics of apolipoprotein B100 (apoB100) and triglyceride metabolism in VLDL in homozygous subjects. In 10 homozygote *TM6SF2* E167K carriers and 10 matched controls, we employed stable-isotope tracer and compartmental modeling techniques to determine apoB100 and triglyceride kinetics in the 2 major VLDL subfractions: large triglyceride-rich VLDL_1_ and smaller, less triglyceride-rich VLDL_2_. VLDL_1_-apoB100 production was markedly reduced in homozygote *TM6SF2* E167K carriers compared with controls. Likewise, VLDL_1_-triglyceride production was 35% lower in the *TM6SF2* E167K carriers. In contrast, the direct production rates for VLDL_2_-apoB100 and triglyceride were not different between carriers and controls. In conclusion, the *TM6SF2* E167K genetic variant was linked to a specific reduction in hepatic secretion of large triglyceride-rich VLDL_1_. The impaired secretion of VLDL_1_ explains the reduced plasma triglyceride concentration and provides a basis for understanding the lower risk of cardiovascular disease associated with the *TM6SF2* E167K genetic variant.

## Graphical abstract

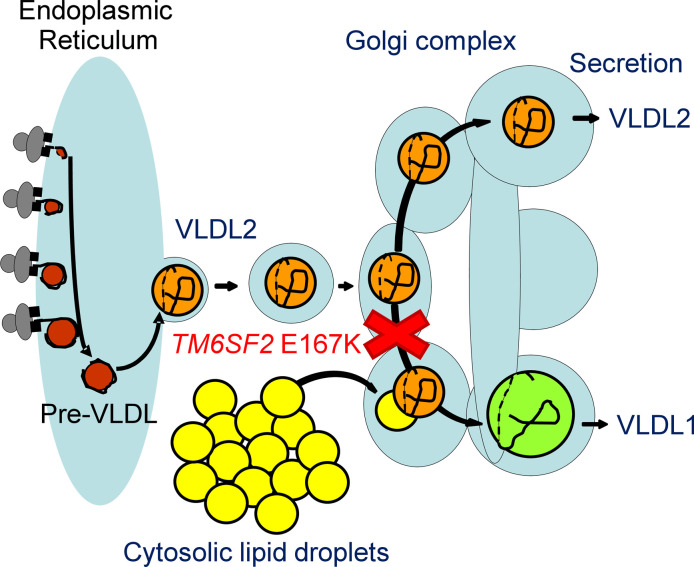

## Introduction

Nonalcoholic fatty liver disease (NAFLD) is a condition defined by excessive (>5% by weight) fat accumulation in the liver. As a consequence of the obesity and diabetes epidemic, NAFLD has become the most common liver disorder in developed countries, with more than 100 million adults and children affected in the United States alone (1). Although NAFLD may progress to severe liver disease, including nonalcoholic steatohepatitis and cirrhosis (2, 3), the most common cause of death in patients with the condition is atherosclerotic cardiovascular disease (4).

NAFLD has a multifactorial etiology that includes a strong genetic component. The transmembrane 6 superfamily member 2 (*TM6SF2*) rs58542926 variant, encoding for a glutamic acid to lysine substitution at position 167 of the amino acid sequence (E167K), has been identified as one of the most important genetic determinants of hepatic fat content (5–7). This single nucleotide variant reduces expression of the TM6SF2 protein by 46% in liver cells (7), and its prevalence is around 7% in Caucasian populations (8). Despite extensive investigation, the molecular function of *TM6SF2* remains unclear. Localized on chromosome 19, the gene is expressed mainly in the liver, small intestine, and kidney (7). It encodes a 351–amino acid protein with 7–10 predicted transmembrane domains (9) that resides in the endoplasmic reticulum (ER) and the ER-Golgi intermediate compartment (ERGIC) (9).

In theory, the E167K variant could induce hepatic fat accumulation by increasing the inflow or synthesis of lipids in the liver (i.e., by stimulating hepatic lipid uptake or hepatic de novo lipogenesis [DNL]), or by blocking lipid catabolism or outflow (i.e., by reducing hepatic β-oxidation or very low-density lipoprotein [VLDL] secretion) (10–16). Of these potential mechanisms, attention has focused most on VLDL metabolism. In an in vitro model *TM6SF2* E167K was associated with reduced hepatic secretion of VLDL triglyceride (TG) (9, 17), and inhibition of *Tm6sf2* expression in mice resulted in increased lipid droplet TG content and impaired secretion of VLDL-TG (9, 17, 18). Interestingly, Smagris et al. observed in *Tm6sf2*-deficient mice no decrease in the number of VLDL particles secreted but a smaller diameter for VLDL (18). These observations raise the possibility that TM6SF2 may regulate hepatic lipidation of nascent VLDL prior to its secretion into the bloodstream (18). This mechanism would also explain why the *TM6SF2* E167K variant associates with lower plasma cholesterol and TG levels in population studies (7, 19). However, other findings in animal and in vitro models complicate the picture and argue against this hypothesis; hepatic overexpression of mouse *Tm6sf2* leads to decreased plasma TG (17), while transient overexpression of human *TM6SF2* in mice results in increased plasma lipid levels (20). Further, expression of *TM6SF2* E167K in vitro has been shown to upregulate genes involved in cholesterol biosynthesis and DNL but to reduce apolipoprotein B100 (apoB100) secretion (21). These conflicting findings — especially the fact that *Tm6sf2* overexpression appears to lead to the same phenotype as *Tm6sf2* deficiency — question how well results from mouse models translate into human physiology in this instance and demonstrate the clear need for clinical metabolic studies to clarify the issue.

Investigations of hepatic lipid metabolism in humans have reported that the *TM6SF2* E167K genetic variant causes relative phosphatidylcholine (PC) deficiency due to impaired synthesis of this lipid from polyunsaturated fatty acids (22). Carriers of the genetic variant have also been shown to have increased cholesteryl esters in the liver and an altered profile of circulating TG species (23). A fundamental gap in our understanding is the impact of *TM6SF2* on plasma lipid and lipoprotein metabolism in humans and its consequences for risk of cardiovascular disease. Of particular importance is the effect of the E167K variant on the rates of synthesis, secretion, and lipolysis of VLDL because these lipoproteins give rise through delipidation and remodeling to the cholesterol-rich lipoprotein species — remnants and LDL — that are implicated in atherogenesis. To address this, we performed for the first time to our knowledge detailed kinetic studies of apoB100 and TG metabolism in VLDL in subjects homozygous for *TM6SF2* E167K to test the hypothesis that the genetic variant affects hepatic VLDL secretion.

## Results

### Phenotypic characterization of study subjects.

We examined homozygote *TM6SF2* E167K carriers (*n* = 10) and control subjects (*n* = 10). The 2 groups were matched for BMI, waist circumference, and indices of glucose homeostasis; measures of visceral (VAT) and abdominal subcutaneous (SAT) adipose tissue also did not differ significantly between the groups (*P* = 0.35 for both). As expected, the *TM6SF2* E167K carriers had more than 2-fold higher liver fat content than noncarriers (mean of 8.4% ± 6.1 vs. 4.0% ± 5.2, respectively, *P* = 0.023) (Table 1).

### Plasma lipoprotein levels and VLDL composition.

Both total plasma apoB (81 ± 19 vs. 106 ± 19 mg/dL; *P* = 0.003) and plasma TG (1.0 ± 0.5 vs. 1.4 ± 0.3 mmol/L; *P* = 0.011) were significantly lower in homozygote *TM6SF2* carriers compared with controls (Table 1). The reduction in plasma TG was mainly attributable to a 50% reduction of the amount of this lipid in VLDL_1_ (Table 1).

**Figure 1:**
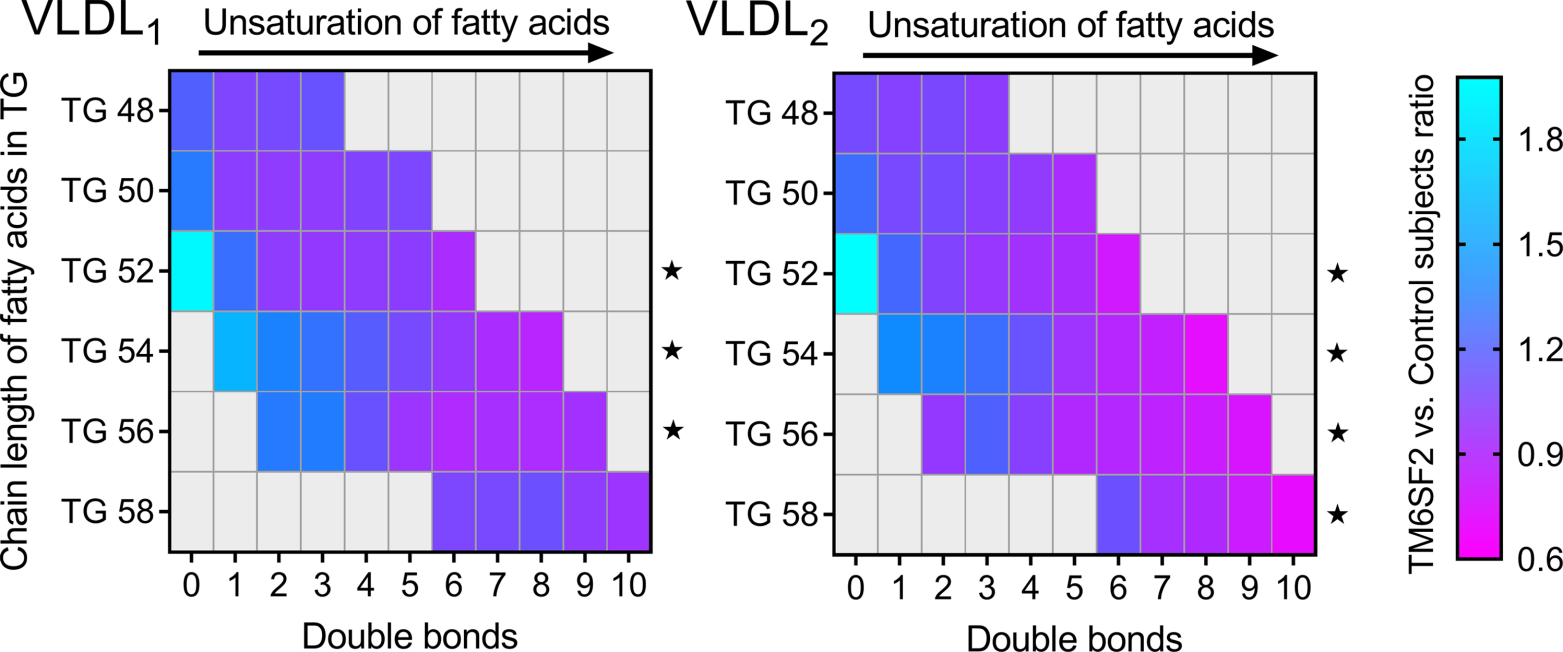
Decrease in the relative abundance (analyzed as mol%) of longer and more unsaturated fatty acids in VLDL_1_ and VLDL_2_ TGs in homozygous carriers of the *TM6SF2* E167K genetic variant. Lipids were extracted from VLDL_1_ and VLDL_2_, and the number of double bonds in fatty acids (i.e., degree of unsaturation) in TGs containing 48 to 58 carbon molecules was determined. The colors indicate the ratio of the individual lipid species in carriers of the *TM6SF2* E167K genetic variant (*n* = 10) versus controls (*n* = 10). A linear mixed model with number of double bonds as fixed effect and subject as random effect was used to test for a linear effect of the number of double bonds, within fixed chain lengths. Data were normalized to mean of control group; *P* values correspond to tests of the linear effect of double bonds differing from 0. *P < 0.05.

Lipidomic analysis of isolated VLDL_1_ and VLDL_2_ showed that the composition of these lipoproteins with respect to major lipid classes did not differ between the 2 subject groups (Table 2). Closer investigation of individual lipid species did, however, reveal differences between the 2 genotypes. In both VLDL_1_ and VLDL_2_ TG, the relative abundance of unsaturated fatty acids in the *TM6SF2* carriers decreased with increased number of double bonds (Figure 1).

### VLDL kinetics.

To test the hypothesis that the *TM6SF2* E167K genetic variant has a specific effect on hepatic VLDL secretion, we measured apoB100 and TG kinetics in the 2 major VLDL subfractions — large TG-rich VLDL_1_ and smaller, less TG-rich VLDL_2_. Results showed that production of VLDL_1_-apoB100, which is reflective of the number of VLDL_1_ particles secreted per day, was markedly reduced in homozygote *TM6SF2* E167K carriers compared with controls (435 ± 200 vs. 788 ± 210 mg/d, *P* = 0.003) (Table 3). Likewise, production of VLDL_1_-TG was 35% lower in the former compared with the latter group (21,307 ± 10,831 vs. 32,774 ± 13,055 mg/d, *P* = 0.043). In contrast, the direct production rates for VLDL_2_-apoB100 and -TG were not different between carriers and noncarriers (Table 3). Fractional catabolic rates (FCRs) for VLDL_1_ and VLDL_2_-apoB100 and -TG did not differ significantly between the groups (Table 3). There were no significant differences in hepatic DNL measured in VLDL_1_ or plasma β-hydroxybutyrate levels (i.e., a marker of hepatic β-oxidation) between the 2 groups of study subjects (Table 1).

Because plasma apoC-III levels are strong predictors of VLDL_1_-TG FCR (24) and there was a tendency to lower levels of this apoprotein in the *TM6SF2* homozygotes, we explored the association of plasma apoC-III levels with VLDL_1_-TG FCR in the 2 subject groups and found that the difference in apoC-III plasma levels did not correlate with the difference in VLDL_1_-FCR (*r* = –0.21, *P* = 0.39).

## Discussion

The aim of the present study was to elucidate further the phenotype associated with the genetic variant *TM6SF2* E167K, in particular how it affects hepatic VLDL secretion, and by extrapolation risk of atherosclerotic cardiovascular disease. Our results show clearly that in subjects homozygous for this variant, there is a specific impact on the synthesis and secretion of large TG-rich VLDL_1_ but no effect on the direct secretion of the smaller, less TG-rich VLDL_2_. This finding is informative since we know from previous studies that production of these 2 VLDL subfractions is regulated independently (25). There was no significant influence of the *TM6SF2* E167K variant on the lipolysis or clearance rates of either VLDL subfraction. The decrease in VLDL_1_ production is the likely proximal cause of the reduction in plasma apoB, although it should be noted that the decrease in VLDL_1_ apoB concentration in *TM6SF2* E167K homozygotes was insufficient to account for the much larger decrease in apoB overall. It is likely that a consequent fall in the levels of the delipidation products of VLDL_1_ — remnants and LDL — contributed to the lower total apoB (25) and also to the reduced risk of atherosclerotic cardiovascular disease associated with this genetic variant (20, 26).

Cell culture studies have revealed that the assembly of VLDL in hepatocytes is a multistage process (27). In the first step, primordial pre-VLDL particles are formed in the ER. These then enter the secretory pathway and are converted to VLDL_2_-sized particles by incremental addition of TG in a stepwise lipidation process (28). The precursor VLDL_2_ particles either are released from the cell (and are detected as “direct” VLDL_2_ apoB secretion in Table 3) or undergo a further lipidation step to become large, TG-rich VLDL_1_, which are then secreted (28, 29). The conversion of VLDL_2_-sized precursor particles to VLDL_1_ occurs in a smooth membrane compartment, likely the ERGIC (30). The process requires bulk addition of TG because VLDL_1_ contains a mean of 43,154 molecules of TG per particle (i.e., per apoB, Table 2) compared with the 11,180 per particle in VLDL_2_. Formation of VLDL_1_ is believed to be mediated by fusion of a lipid droplet formed in the smooth ER with the VLDL_2_-sized precursor (31).

Thus, assembly of TG-rich VLDL_1_ is highly dependent on the availability of TGs to form these smooth ER lipid droplets, which in turn requires a ready supply of TG within the hepatocyte (32). This can come from DNL, fatty acid uptake from the circulation, or mobilization of cytoplasmic lipid stores (macroscopic lipid droplets) (33). The last is a major source, and VLDL_1_ secretion is a way in which the liver can regulate the amount of stored intracellular TG (33, 34). In most subject groups it is possible to observe an association between the amount of TG in intracellular stores (liver fat) and the secretion rates of VLDL_1_ apoB100 and TG (34). Inheritance of the *TM6SF2* E167K variant disrupts this relationship; liver fat increases but VLDL_1_ secretion is reduced.

Given the information available from this and earlier studies, it is reasonable to postulate that *TM6SF2* which is located in the ER/ERGIC region (and hence adjacent to the lipoprotein assembly and secretion pathway) may be critical for the formation of lipid droplets that fuse with VLDL_2_-sized precursor particles to form VLDL_1_ or be required for the fusion process per se. The mechanism responsible for the generation of lipid droplets within the ER is not completely clear. It is known that enzymes capable of synthesizing neutral lipids are present in this cellular location (35–37), and once formed, small primordial droplets increase in size as they pass through the smooth ER, either by individual expansion or by fusion with other droplets (33, 36, 37). The availability of phospholipids, especially PC, is considered an important factor in facilitating the increase in size of lipid droplets (38). Krahmer et al. demonstrated that expanding lipid droplets recruit CTP:phosphocholine cytidylyltransferase, the rate-limiting enzyme for PC synthesis, and that this enzyme is activated when it binds to the surface of the droplets (38). Luukkonen et al. demonstrated that the *TM6SF2* E167K genetic variant leads to altered hepatic lipid metabolism, resulting in relative PC deficiency due to impaired synthesis of this lipid from polyunsaturated fatty acids (22). In accord with this finding, we observed significant decrease in the relative abundance of longer and more unsaturated fatty acids in VLDL_1_ and VLDL_2_ TGs in homozygous carriers of the *TM6SF2* E167K genetic variant. Thus, it is possible that PC deficiency due to inheritance of the *TM6SF2* E167K variant leads to an assembly defect and failure to synthesize and secrete large TG-rich VLDL particles from the liver. However, the observation about fatty acid length and saturation was observed in both large TG-rich VLDL_1_ and smaller VLDL_2_ fractions. This finding argues against a specific effect on the assembly of large VLDL_1_ particles.

It is also possible that the impact of the genetic variant is secondary and caused by other genes that are coexpressing with *TM6SF2* as proposed by Luukkonen et al. (22). This action has been suggested to explain the reduced hepatic expression of apoC-III in subjects with the *TM6SF2* E167K variant (22). ApoC-III has a number of roles in VLDL metabolism. Pertinent to the present discussion is the proposal that this apolipoprotein may exert its effects on lipid substrate utilization for the second-step VLDL assembly process (39). Very recently, Li et al. reported that *TM6SF2* stabilizes apoB by interacting with ER lipid raft protein 1 and 2, which mediates the ER-associated degradation of inositol 1,4,5-trisphosphate receptors (40). However, the relevance of these findings for human pathophysiology is still unclear, and it is not evident how defective apoB stabilization would specifically impair hepatic VLDL_1_ secretion.

In conclusion, here we demonstrate that the *TM6SF2* E167K genetic variant is linked to a specific reduction in hepatic secretion of VLDL_1_. The proposed underlying mechanism is a relative failure to secrete large, TG-rich lipoproteins, and the inability to export lipid efficiently through the VLDL pathway is a satisfactory explanation for the accumulation of liver fat. The 35% reduction in flow of apoB down the delipidation cascade will have predictable consequences for the products of lipolysis of VLDL_1_ — remnant VLDL particles and LDL — which are implicated as causal factors in atherosclerosis (25). These metabolic perturbations explain the reduced plasma TG concentrations (7, 26, 41), and lower risk of cardiovascular disease (20, 26) associated with the *TM6SF2* E167K genetic variant.

## Methods

### Subjects.

A group of 20 subjects, recruited on the basis of their *TM6SF2* E167K and *PNPLA3* I143M genotypes, participated in this study. Twelve subjects, identified from the THL Biobank (a country-wide biobank that collects and stores research samples from all over Finland, https://thl.fi/en/web/thl-biobank/about-thl-biobank), had participated in earlier studies where exome sequencing or genotyping and imputation had been performed to explore genes involved in lipid metabolism. The remaining 8 subjects came from previously examined kinetic study cohorts (24, 42, 43). All had given oral consent that allowed them to be invited to further studies focused on lipid metabolism. Two groups were included in this study: 10 homozygote carriers for *TM6SF2* E167K (9 men and 1 woman) and 10 control subjects (9 men and 1 woman), matched for degree of obesity and glycemic status, who were not carriers of *TM6SF2* E167K and also were not homozygous for *PNPLA3* I143M (Table 1). Inclusion criteria were age 18–70 years, nonsmoking status, and BMI < 33 kg/m^2^. Exclusion criteria included a history of cardiovascular or other severe disease, any condition affecting lipid levels, abnormalities in thyroid or kidney function, liver disease other than NAFLD, abnormal blood count, glycated hemoglobin (HbA1c) > 42 mmol/mol, LDL-cholesterol > 4.5 mmol/L, plasma TG > 2.5 mmol/L, uncontrolled hypertension (>160 mmHg systolic and /or >105 mmHg diastolic blood pressure), use of thiazide diuretics (>25 mg/d), use of medications affecting lipid or glucose metabolism, or high alcohol consumption (>30 g/d for men and >20 g for women). The participants were asked to abstain from alcohol and strenuous physical exercise during leisure time before metabolic investigations.

### Study design.

The kinetic studies were performed as described previously (24, 44). On the evening before the VLDL apoB100/TG kinetic study, ^2^H_2_O (2 g/kg) was given between 1800 and 2200 hours to give an assessment of the degree of the contribution of hepatic DNL to VLDL_1_ TGs (see refs. 45, 46). The subjects came back at 7:30 am to the research unit of the Helsinki University Hospital after a 12-hour overnight fast. An indwelling cannula was inserted into an antecubital vein for blood sampling, and a second cannula was inserted into the opposite antecubital vein. The subjects received a bolus injection of [^2^H_5_]-glycerol (500 mg) and [^2^H_3_]-leucine (7 mg/kg). Blood samples were drawn before tracer injection and at frequent intervals thereafter (24, 44).

### Lipoprotein isolation.

VLDL_1_ and VLDL_2_ were separated from plasma as described previously (44). ApoB100 was obtained from the 2 VLDL subfractions and hydrolyzed, derivatized, and subjected to gas chromatography mass spectrometry to measure tracer leucine enrichment (44). Likewise, TG was extracted from the VLDL fractions and the tracer glycerol enrichment determined (44). The particle composition and apoB mass of VLDL_1_ and VLDL_2_ were determined before and at 4 and 8 hours after tracer injection. The subjects continued to fast until 5 pm, when the last blood sample was taken and they were served a standard dinner.

### Biochemical analyses.

Fasting TG and cholesterol concentrations in total plasma and in the VLDL_1_ and VLDL_2_ fractions, and HDL-cholesterol and LDL-cholesterol, were analyzed by automated enzymatic methods using the Konelab 60i analyzer (Thermo Fisher Scientific). Concentrations of glucose (Gluco-quant, Roche Diagnostics) and insulin (electrochemiluminescence with Roche sandwich immunoassay using Cobas autoanalyzer) were measured in fasting blood samples and HOMA-IR was calculated (47). Plasma levels of apoC-III were measured immune-turbidometrically (Kamiya Biochemical Company) and β-hydroxybutyrate concentrations by using a β-hydroxybutyrate FS kit (Diagnostic Systems) on a Konelab 60i analyzer (Thermo Fisher Scientific). Plasma fatty acids were analyzed using an automatic enzymatic colorimetric method (Wako Chemicals).

### Lipidomics.

Lipid extraction of 50 μL of each VLDL fraction was performed using the BUME method (48). TGs were quantified by direct infusion (shotgun) analysis on a QTRAP 5500 mass spectrometer (SCIEX) equipped with a robotic nanoflow ion source, TriVersa NanoMate (Advion BioSciences). The analysis was performed in positive ion mode by neutral loss detection of 10 common acyl fragments formed during collision-induced dissociation according to previous work (49). Glyceryl-d_5_-hexadecanoate (CDN Isotopes) was added during the extraction and used for quantification.

### Imaging.

Liver fat content was measured using proton magnetic spectroscopy (1.5 T whole-body device) (34), and subcutaneous and visceral fat were measured by magnetic resonance imaging (50). All imaging analyses were performed by a single person. Subjects were advised to fast for 4 hours before scans were taken.

### Genotyping.

The genotypes of participants were confirmed upon recruitment to the present study. DNA was extracted from blood using DNeasy Blood & Tissue Kit (Qiagen) and used to determine *TM6SF2* rs58542926 (E167K) and *PNPLA3* rs738409 (I148M) status by TaqMan assays (Life Technologies, Thermo Fisher Scientific) using CFX384 Real Time PCR detection system (Bio-Rad Laboratories). Data analysis was performed using Bio-Rad CFX manager software.

### Modeling.

The multicompartmental model used to analyze simultaneously VLDL apoB100 and TG kinetics was constructed using SAAM II (The Epsilon Group) (51). Inputs to the model were (a) injected amounts of [^2^H_3_] leucine and [^2^H_5_] glycerol, (b) apoB100 and TG pool sizes in VLDL_1_ and VLDL_2_, (c) enrichment curve of plasma leucine, (d) enrichment of deuterated leucine in in VLDL_1_- and VLDL_2_-apoB100, and (e) enrichment of deuterated glycerol in VLDL_1_- and VLDL_2_-TG (44). Outputs were production (in mg/d) and fractional clearance rates (in pools/d) for apoB100 and TG for each subfraction and the transfer rates from VLDL_1_ to VLDL_2_.

### Statistics.

Statistical calculations were performed using R (version 3.6.3). Data are reported as mean ± SD, and *P* values were calculated using the Kruskal-Wallis test. Correlation coefficients refer to the Spearman’s rank correlation coefficient. Trend analyses for lipidomics data were performed using linear mixed models with number of double bonds as fixed effect and subject as random effect. Data were normalized to mean of control group; *P* values correspond to tests of the linear effect of double bonds differing from 0. *P* < 0.05 was considered statistically significant.

### Study approval.

The study protocol was approved by the Ethics Committee of Helsinki University Central Hospital, Helsinki, Finland. ClinicalTrials.gov Identifier: NCT04209816. The study was performed in accordance with the Declaration of Helsinki and the European Medicines Agency Note for guidance on good clinical practice. All study participants signed a written informed consent form before any study procedures were initiated.

## Author contributions

The authors contributed to the present work as follows: JB, NM, and MRT contributed to conception and design; MA, EB, NM, SS, JR, MS, PR, S Ripatti, AP, RMM, AH, and S Romeo to the acquisition of data or analysis; and JB, MA, EB, CJP, and MRT to the interpretation of data. JB, MA, CJP, and MRT drafted the original and revised manuscripts, and all authors approved the final version to be published.

## Figures and Tables

**Table 1:**
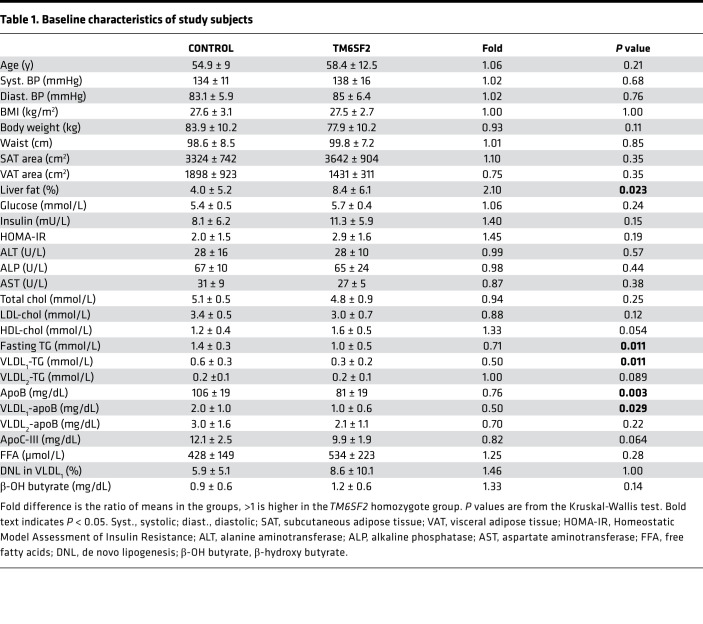
Baseline characteristics of study subjects

**Table 2:**
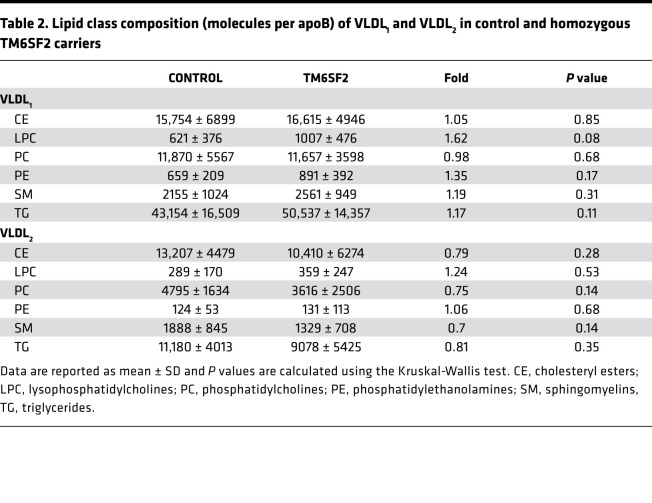
Lipid class composition (molecules per apoB) of VLDL_1_ and VLDL_2_ in control and homozygous TM6SF2 carriers

**Table 3:**
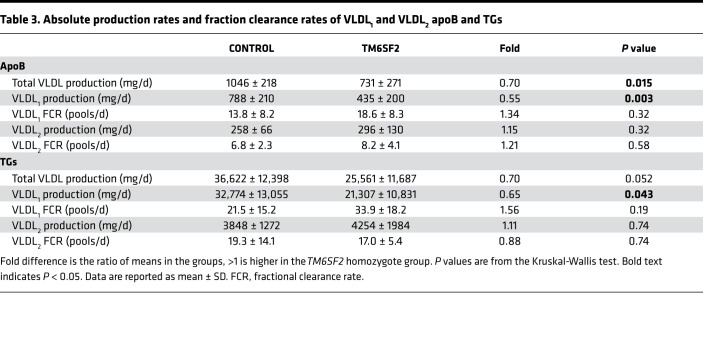
Absolute production rates and fraction clearance rates of VLDL_1_ and VLDL_2_ apoB and TGs

## Version 1 11/10/2020

In-Press Preview

## Version 2 12/17/2020

Electronic publication
